# SARS-CoV-2 and Guillain-Barré syndrome: molecular mimicry with human heat shock proteins as potential pathogenic mechanism

**DOI:** 10.1007/s12192-020-01145-6

**Published:** 2020-07-29

**Authors:** Guglielmo Lucchese, Agnes Flöel

**Affiliations:** 1 Department of Neurology, Universitätsmedizin Greifswald, Ferdinand-Sauerbruch-Str, 17475 Greifswald, Germany; 2 German Center for Neurodegenerative Diseases, Rostock/Greifswald, Greifswald, Germany

**Keywords:** COVID-19, Neuropathy, Demyelination

## Abstract

Severe acute respiratory syndrome-related coronavirus 2 infection has been associated with Guillain-Barré syndrome. We investigated here the potential mechanism underlying the virus-induced damage of the peripheral nervous systems by searching the viral amino acid sequence for peptides common to human autoantigens associated with immune-mediated polyneuropathies. Our results show molecular mimicry between the virus and human heat shock proteins 90 and 60, which are associated with Guillain-Barré syndrome and other autoimmune diseases. Crucially, the shared peptides are embedded in immunoreactive epitopes that have been experimentally validated in the human host.

**Electronic supplementary material:**

The online version of this article (10.1007/s12192-020-01145-6) contains supplementary material, which is available to authorized users.

## Introduction

The disease (COVID-19) caused by the severe acute respiratory syndrome-related coronavirus 2 (SARS-CoV-2) encompasses a broad array of symptoms and complications including most commonly fever, coughing, dyspnea, pneumonia, and respiratory failure, as well as less frequently myalgia, arthralgia, skin lesions, diarrhea, nausea, vomiting, and renal failure (Chen et al. 2020; Guan et al. 2020). Additionally, neurological involvement in the form of anosmia, ageusia, headache, nausea and vomiting, seizures, encephalopathy, and ischemic stroke has been described (Vaira et al. 2020; Asadi-Pooya and Simani 2020), possibly even as an indicator of unfavorable prognosis. In a retrospective study, it was found that 22% of the patients who died presented with disorders of consciousness at admission compared with 1% who recovered (Chen et al. 2020).

However, the exact pathogenesis of COVID-19-related neurological damage is still largely unknown, and diverse mechanisms might play a role. Neurotropism of coronaviruses is well known, and SARS-CoV-2 and SARS-CoV, among others, are not confined to the respiratory tract but can also invade the central nervous system (Li et al. 2020). At the same time, evidence is mounting that COVID-19 is associated with immune-mediated neurological complications, for example, in the form of Guillain-Barré syndrome (GBS) (Toscano et al. 2020; Coen et al. 2020). Indeed, neurological sequelae of infections are a well described phenomenon, and previous viral epidemic outbreaks have already shown that immune-mediated mechanisms may induce damage to the nervous system and specifically GBS (Cao-Lormeau et al. 2016; Lucchese and Kanduc 2016), which is a classical example of molecular mimicry (Dalakas et al. 2015). Among other mechanisms, molecular mimicry between SARS-CoV-2 and various human organs and tissues has been already postulated as possible trigger of multi-organ autoimmunity in COVID-19 (Cappello 2020; Angileri et al. 2020a, b).

We tested here the hypothesis that neuropathy in COVID-19 might be the consequence of molecular mimicry between the SARS-CoV-2 and human autoantigens involved in inflammatory polyneuropathies by analyzing the peptide sharing between the virus and such protein antigens.

## Materials and methods

A set formed by the primary amino acid (aa) sequences of 41 human protein antigens associated with acute (GBS; Miller Fisher Syndrome) and chronic (chronic inflammatory demyelinating polyneuropathy, CIDP; multifocal motor neuropathy, MMN) immune-mediated neuropathies was retrieved from the UniProt database (Table 1; www.uniprot.org, Magrane et al. 2011).

**Table 1:** Protein antigens associated with acute and chronic immune-mediated neuropathies (UniProt-ID, Acronym, Name, Gene)

O94856-8 NFASC Isoform 8 (NF155) of Neurofascin GN=NFASC (Querol et al. 2017; Burnor et al. 2018)	
O94856-4 NFASC Isoform 4 (NF140) of Neurofascin GN=NFASC (Burnor et al. 2018)	
O94856-1 NFASC Neurofascin (NF186) GN=NFASC (Burnor et al. 2018)	
P05455 LA Lupus La protein GN=SSB (Querol et al. 2017)	
P07900 HS90A Heat shock protein HSP 90-alpha GN=HSP90AA1 (Yonekura et al. 2004)	
P08238 HS90B Heat shock protein HSP 90-beta GN=HSP90AB1 (Yonekura et al. 2004)	
P0DMV8 HS71A Heat shock 70 kDa protein 1A GN=HSPA1A (Yonekura et al. 2004)	
P10155 RO60 60 kDa SS-A/Ro ribonucleoprotein GN=RO60 (Yonekura et al. 2004)	
P10809 CH60 60 kDa Heat shock protein, mitochondrial GN=HSPD1 (Yonekura et al. 2004)	
P11142 HSP7C Heat shock cognate 71 kDa protein GN=HSPA8 (Yonekura et al. 2004)	
P14625 ENPL Endoplasmin GN=HSP90B1 (Yonekura et al. 2004)	
P17066 HSP76 Heat shock 70 kDa protein 6 GN=HSPA6 (Yonekura et al. 2004)	
P20916 MAG Myelin-associated glycoprotein GN=MAG (Querol et al. 2017)	
P22607 FGFR3 Fibroblast growth factor receptor 3 GN=FGFR3 (Querol et al. 2017)	
P26038 MOES Moesin GN=MSN (Sawai et al. 2014)	
P26378 ELAV4 ELAV-like protein 4 GN=ELAVL4 (Querol et al. 2017)	
P34931 HS71L Heat shock 70 kDa protein 1-like GN=HSPA1L (Yonekura et al. 2004)	
P34932 HSP74 Heat shock 70 kDa protein 4 GN=HSPA4 (Yonekura et al. 2004)	
P38646 GRP75 Stress-70 protein, mitochondrial GN=HSPA9 (Yonekura et al. 2004)	
P48741 HSP77 Putative heat shock 70 kDa protein 7 GN=HSPA7 (Yonekura et al. 2004)	
P54652 HSP72 Heat shock-related 70 kDa protein 2 GN=HSPA2 (Yonekura et al. 2004)	
P61604 CH10 10 kDa Heat shock protein, mitochondrial GN=HSPE1 (Yonekura et al. 2004)	
P78357 CNTP1 Contactin-associated protein 1 GN=CNTNAP1 (Querol et al. 2017)	
Q0VDF9 HSP7E Heat shock 70 kDa protein 14 GN=HSPA14 (Yonekura et al. 2004)	
Q12860 CNTN1 Contactin-1 GN=CNTN1 (Querol et al. 2017)	
Q12926 ELAV2 ELAV-like protein 2 GN=ELAVL2 (Querol et al. 2017)	
Q12988 HSPB3 Heat shock protein beta-3 GN=HSPB3 (Yonekura et al. 2004)	
Q14576 ELAV3 ELAV-like protein 3 GN=ELAVL3 (Querol et al. 2017)	
Q15717 ELAV1 ELAV-like protein 1 GN=ELAVL1 (Querol et al. 2017)	
Q16543 CDC37 Hsp90 co-chaperone Cdc37 GN=CDC37 (Yonekura et al. 2004)	
Q58FF3 ENPLL Putative endoplasmin-like protein GN=HSP90B2P (Yonekura et al. 2004)	
Q58FF6 H90B4 Putative heat shock protein HSP 90-beta 4 GN=HSP90AB4P (Yonekura et al. 2004)	
Q58FF7 H90B3 Putative heat shock protein HSP 90-beta-3 GN=HSP90AB3P (Yonekura et al. 2004)	
Q58FF8 H90B2 Putative heat shock protein HSP 90-beta 2 GN=HSP90AB2P (Yonekura et al. 2004)	
Q58FG0 HS905 Putative heat shock protein HSP 90-alpha A5 GN=HSP90AA5P (Yonekura et al. 2004)	
Q58FG1 HS904 Putative heat shock protein HSP 90-alpha A4 GN=HSP90AA4P (Yonekura et al. 2004)	
Q6ZMI3 GLDN Gliomedin GN=GLDN (Querol et al. 2017)	
Q7L3B6 CD37L Hsp90 co-chaperone Cdc37-like 1 GN=CDC37L1 (Yonekura et al. 2004)	
DPYL5 Dihydropyrimidinase-related protein 5 GN=DPYSL5 (Querol et al. 2017)	
HPBP1 Hsp70-binding protein 1 GN=HSPBP1 (Yonekura et al. 2004)	
CNTP2 Contactin-associated protein-like 2 GN=CNTNAP2 (Querol et al. 2017)	

The entire primary aa sequence of the SARS-CoV-2 was retrieved from https://www.ncbi.nlm.nih.gov/nuccore/MN908947 and dissected into hexapeptides overlapping by 5 residues (for instance, MESLVP, ESLVPG, SLVPGF, and so forth) for a total of *n* = 9649.

Then, each viral peptide was analyzed for occurrences in the set of neuropathy-related protein antigens obtained as described above. The SARS-CoV-2 hexamers were analyzed for occurrences in a set of human dentin-related proteins as negative control (chosen as instance of human tissue not affected by COVID-19). The set was retrieved by searching for “dentin” in the UniProt database and consists of 86 proteins that are listed in the Supplementary Table S1. The analyses were carried out with custom scripts for the MATLAB programming environment.

The Immune Epitope Database (IEDB; www.iedb.org) resource was used to explore the immunological relevance of the shared motifs (Vita et al. 2015).The IEDB was searched for linear epitopes with reported positive T and/or B cell assays in the human host and containing the hexapeptides shared by SARS-CoV-2 and the GBS-related protein antigens. The IEDB is a curated immunological repository containing epitopes that have been experimentally validated as immuno-positive.

## Results

Sequence analysis of the 41 human proteins associated with acute and chronic immune-mediated neuropathies (Table 1) showed that SARS-CoV-2 shares two immunologically relevant hexapeptides (KDKKKK and EIPKEE) with the human heat shock proteins 90 (HSP90B and HSP90B2) and 60 (HSP60), respectively (Table 2). The former hexapeptide is part of 5 experimentally validated epitopes from the SARS-CoV, as catalogued in the IEDB; the latter is part of 1 experimentally validated autoimmune epitope recognized by lymphomononuclear cells of multiple sclerosis (MS) patients (Ruiz-Vázquez and de Castro 2003).

**Table 2:** Hexapeptides of immunologic relevance shared between SARS-CoV-2 and GBS-related proteins as catalogued in the Immune Epitope Database (IEDB; www.iedb.org)12

Shared 6-mer	SARS-CoV-2 protein	Human proteins [UniProt-ID; Gene]	Epitopes [IEDB-ID; Protein; Organism]
KDKKKK	Nucleocapsid Phosphoprotein	Heat shock protein 90-beta [P08238; HSP90AB1] Putative heat shock protein 90-beta 2 [Q58FF8; HSP90AB2P]	KDKKKKTDEAQPLPQRQKKQ [30186; Nucleoprotein; SARS-CoV] EPKKDKKKKTDEAQPL [13680; Nucleoprotein; SARS-CoV] KTFPPTEPKKDKKKK [33669; Nucleoprotein; SARS-CoV] TEPKKDKKKKTDEAQPLPQRQKK + ACET(T1) [63494; Nucleoprotein; SARS-CoV] YKTFPPTEPKKDKKKK [74517; Nucleoprotein; SARS-CoV]
EIPKEE	Orf1ab Polyprotein	60 kDa heat shock protein, mitochondrial [P10809; HSPD1]	VVTEIPKEEKDPGM [112717; 60 kDa heat shock protein; *Homo sapiens*]

Each row presents one shared hexamer (first column), the SARS-CoV-2 protein (second column), the human GBS-related protein(s) (third column), and the experimentally validated immunogenic epitopes containing the same hexamer (last column)

No sharing of immunologically relevant hexapeptides was found between SARS-CoV-2 and the human dentin-related proteins. More details can be found in the supplementary material.

## Discussion

We show in the present study that the SARS-CoV-2 shares aa sequences of proven immunologic potential with the human heat shock proteins (HSPs). HSPs have also been involved in a number of immune-mediated clinical conditions, and they can become the target of immune response, possibly as a consequence of molecular mimicry (Moudgil et al. 2013).

Important from a neurological perspective, autoantibodies targeting different families of HSPs have been shown to be elevated in serum and cerebrospinal fluid (CSF) of patients affected by myasthenia gravis, MS, and, crucially, GBS (Romi et al. 2011). The sharing of peptide motifs with immunologic potential, as demonstrated by their presence within human experimentally validated epitopes, between the virus and HSPs, therefore strongly supports an immune-mediated neurological damage in COVID-19.

In particular, the hexapeptide shared with the HSP90B and HSP90B2 is part of 5 experimentally validated epitopes from the SARS-CoV, as catalogued in the IEDB. Crucially, the hexapeptide is located proximally (epitope-ID: 30186) in the middle (epitope-ID: 13680; 63494) and terminally (epitope-ID: 33669; 74517) in these epitopes (see Table 2), thus constituting the only aa sequence common to all of the epitopes. It is therefore highly likely that the shared hexapeptide is the immunogenic determinant of all the five epitopes. This hexapeptide thus constitutes the ideal candidate to elicit an autoimmune response against HS90B and H90B2 as a consequence of SARS-CoV2 infection.

The second immunologically relevant motif shared by the SARS-CoV-2 and human HSPs belongs to the chaperone protein 60 and, interestingly, has been shown to be recognized by lympho-monocytes of patients affected by demyelinating disease of the central nervous systems (CNS; Ruiz-Vázquez and de Castro 2003). This finding warrants further investigation of a possible association of SARS-CoV-2 infection with inflammation and demyelination not only in the PNS but also in the CNS, which appears to be supported by preliminary clinical reports (Zanin et al. 2020).

HSP60 is a mitochondrial protein that is normally not exposed on the plasma membrane. Nevertheless, immune reactions against intracellular autoantigens are a well-known phenomenon (Greenlee et al. 2015; Racanelli et al. 2011). Moreover, Cappello et al. (2020) postulated that posttranslational modifications (PTMs) to HSPs could induce protein translocation to plasma membrane, and indeed HSP60 localization to the plasma membrane after PTMs has been described (Caruso Bavisotto et al. 2020). Multiple concurrent mechanisms could then explain immune reactions against HSP60 in case of autoimmunity.

In sum, the present data point to immunological targeting of the HSPs 90B, 90B2, and 60 as a potential pathogenic mechanism of neuropathy after SARS-CoV-2 infection and suggest to specifically test sera and CSF of COVID-19 patient affected by GBS and possibly other peripheral neuropathies for autoantibodies against these proteins. Moreover, this data add up to previous literature (Lucchese & Flöel 2020, Lucchese 2020, Cappello et al. 2020) bearing relevance for potential immunomodulatory therapy as well as passive and active immunization in COVID-19.

## Electronic supplementary material

ESM 1 (DOCX 483 kb)
