# Supplementary material for: SARS-CoV-2 and Guillain-Barré syndrome: molecular mimicry with human heat shock proteins as potential pathogenic mechanism
Source: Cell Stress Chaperones. 2020 Jul 29;25(5):731–5. doi: 10.1007/s12192-020-01145-6 (PMC7387880; doi:10.1007/s12192-020-01145-6)
Supplement: Supplementary file 1 — (DOCX 483 kb) [file 12192_2020_1145_MOESM1_ESM.docx]

Table S1. Hexapeptide sharing between SARS-CoV-2 and human proteins related to immune-mediated neuropathies. Hexapeptides belonging to immuno-reactive epitopes as catalogued in the IEDB (immune epitope database) in red.

| 6-mer | SARS-CoV-2  protein | Protein antigens associated with acute and chronic  immune-mediated neuropathies |
| --- | --- | --- |
| KDKKKK | nucleocapsid  phosphoprotein | P08238 HS90B Heat shock protein HSP 90-beta GN=HSP90AB1  Q58FF8 H90B2 Putative heat shock protein HSP 90-beta 2  GN=HSP90AB2P |
| EIPKEE | orf1ab polyprotein | P10809 CH60_HUMAN 60 kDa heat shock protein, mitochondrial  GN=HSPD1 |
| LNGVTL | orf1ab polyprotein | P78357 CNTP1 Contactin-associated protein 1 GN=CNTNAP1 |
| LPYPDP | orf1ab polyprotein | P34932 HSP74 Heat shock 70 kDa protein 4 GN=HSPA4 |
| PNDDTL | orf1ab polyprotein | Q6ZMI3 GLDN Gliomedin GN=GLDN |
| RAKVTS | orf1ab polyprotein | Q9UHC6 CNTP2 Contactin-associated protein-like 2 GN=CNTNAP2 |
| SYYSLL | orf1ab polyprotein | P78357 CNTP1 Contactin-associated protein 1 GN=CNTNAP1 |

Table S2. Hexapeptide sharing between SARS-CoV-2 and human dentin-related proteins.

No hexpeptides belong to any immuno-reative epitope as catalogued in the IEDB (immune epitope database)

| 6-mer | SARS-CoV-2  protein | Dentin-related protein |
| --- | --- | --- |
| AAGLEA | ORF3a protein | O15230 LAMA5 Laminin subunit alpha-5 GN=LAMA5 |
| AEAELA | orf1ab polyprotein | O15230 LAMA5 Laminin subunit alpha-5 GN=LAMA5 |
| AENSVA | surface glycoprotein | Q15465 SHH Sonic hedgehog protein GN=SHH |
| CIDGAL | orf1ab polyprotein | O75581 LRP6 Low-density lipoprotein receptor-related protein 6  GN=LRP6 |
| EEKFKE | orf1ab polyprotein | P78562 PHEX Phosphate-regulating neutral endopeptidase PHEX  GN=PHEX |
| EGSSVE | orf1ab polyprotein | Q6UB99 ANR11 Ankyrin repeat domain-containing protein 11  GN=ANKRD11 |
| EVLTEE | orf1ab polyprotein | P24821 TENA Tenascin  GN=TNC |
| GPPGTG | orf1ab polyprotein | O75351 VPS4B Vacuolar protein sorting-associated protein 4B  GN=VPS4B |
| GVVTTV | orf1ab polyprotein | P08138 TNR16 Tumor necrosis factor receptor  superfamily member 16 GN=NGFR |
| PAQLPA | orf1ab polyprotein | Q9C0K0 BC11B B-cell lymphoma/leukemia 11B GN=BCL11B |
| PGTGKS | orf1ab polyprotein | O75351 VPS4B Vacuolar protein sorting-associated protein 4B  GN=VPS4B |
| PIHSLR | orf1ab polyprotein | O75581 LRP6 Low-density lipoprotein receptor-related protein 6  GN=LRP6 |
| PPGTGK | orf1ab polyprotein | O75351 VPS4B Vacuolar protein sorting-associated protein 4B  GN=VPS4B |
| SGLKTI | orf1ab polyprotein | Q14524 SCN5A Sodium channel protein type 5 subunit alpha  GN=SCN5A |
| VLTEEV | orf1ab polyprotein | P24821 TENA Tenascin GN=TNC |
| VVTTVM | orf1ab polyprotein | P08138 TNR16 Tumor necrosis factor receptor  superfamily member 16 GN=NGFR |
